# A Case for Using Genomics and a Bioinformatics Pipeline to Develop Sensitive and Species-Specific PCR-Based Diagnostics for Soil-Transmitted Helminths

**DOI:** 10.3389/fgene.2019.00883

**Published:** 2019-09-23

**Authors:** Jessica R. Grant, Nils Pilotte, Steven A. Williams

**Affiliations:** ^1^Department of Biological Sciences, Smith College, Northampton, MA, United States; ^2^Molecular and Cellular Biology Program, University of Massachusetts, Amherst, MA, United States

**Keywords:** soil-transmitted helminth, molecular diagnostics, DNA diagnostics, polymerase chain reaction (PCR), quantitative PCR

## Abstract

The balance of expense and ease of use vs. specificity and sensitivity in diagnostic assays for helminth disease is an important consideration, with expense and ease often winning out in endemic areas where funds and sophisticated equipment may be scarce. In this review, we argue that molecular diagnostics, specifically new assays that have been developed with the aid of next-generation sequence data and robust bioinformatic tools, more than make up for their expense with the benefit of a clear and precise assessment of the situation on the ground. Elimination efforts associated with the London Declaration and the World Health Organization (WHO) 2020 Roadmap have resulted in areas of low disease incidence and reduced infection burdens. An accurate assessment of infection levels is critical for determining where and when the programs can be successfully ended. Thus, more sensitive assays are needed in locations where elimination efforts are approaching a successful conclusion. Although microscopy or more general PCR targets have a role to play, they can mislead and cause study results to be confounded. Hyper-specific qPCR assays enable a more definitive assessment of the situation in the field, as well as of shifting dynamics and emerging diseases.

## Introduction

Parasitic worms impact the health and economic well-being of billions of people worldwide. Soil-transmitted helminths (STH) are a burden in the tropics and subtropics and contribute to an estimated 1.9 to 2.1 million disability-adjusted life years (DALYs) and US $7.5 billion to US $138.9 billion in loss of productivity (Bartsch et al., 2016; Kyu et al., 2018). Efforts are underway to eliminate STH, with the ambitious goal of controlling morbidity by the year 2020 (Becker et al., 2018; Uniting to Combat NTDs). Mass drug administrations (MDA) and water, sanitation and hygiene (WASH) programs across endemic countries are making headway (Hicks et al., 2015; Truscott et al., 2016; Weatherhead et al., 2017; Truscott et al., 2019), but with 2020 fast approaching, there are still many challenges to reaching this goal. An important concern is where to enact and when to cease MDA. This depends on accurately mapping the current burden in communities (2018 Action Group Meeting). Sensitive, species-specific diagnostics are critical to properly evaluating the success of these programs, as well as addressing where to focus efforts and when interventions can be ended (Weatherhead et al., 2017).

Diagnostic techniques need to be inexpensive, practical, and give consistent results across technicians and laboratories. Importantly, they must be accurate, sensitive, and easily interpreted. Microscopy has long been relied on as the standard for diagnosis of intestinal parasites, including soil transmitted helminths (Beaver and Martin, 1968). Several copromicroscopic methods are in use, including FLOTAC (Cringoli et al., 2010), MINI-FLOTAC (Maurelli et al., 2014), several modifications of the McMaster technique (Mines, 1977), and Kato-Katz (Katz et al., 1972) (KK). Of these, KK is the most commonly used for STH diagnosis because it is relatively easy to perform in the field and is generally more sensitive than other microscopic methods (Moser et al., 2018). With any of these tests, even highly trained microscopists can misidentify species or give inconsistent results (Krauth et al., 2012), and they are notoriously insensitive in regions with low infection rates (Nikolay et al., 2014; Buonfrate et al., 2015; Speich et al., 2015; Acosta Soto et al., 2017).

Molecular diagnostics have been garnering more interest in the last few years, as their superior sensitivity has been proven and their acceptance by the research community has increased (Easton et al., 2016; Halfon et al., 2017; Holt et al., 2017). However, as with any advance, there are technical problems to overcome. For example, DNA extraction efficiency and preservation of samples prior to testing will affect diagnostic reliability (Andersen et al., 2013; Sarhan et al., 2015; Hidalgo et al., 2018; Papaiakovou et al., 2018). Notably, *Trichuris trichiura* eggs are notoriously difficult to break open, and this impacts the sensitivity of molecular assays, but techniques are being developed and improved to the point where consistently good results are achievable (Harmon et al., 2006; Nunes et al., 2006; Kaisar et al., 2017). Although molecular diagnostics are not inexpensive, microscopy techniques are also expensive and can be difficult to scale up, whereas the costs of qPCR have the potential to decrease, as studies show that multiple technical replicates may not be crucial and other cost-cutting measures, such as cheaper, more effective sample preservation and pooling are explored (Easton et al., 2017; Papaiakovou et al., 2018; Truscott et al., 2019). Until recently, most PCR-based assays have targeted well-characterized and conserved regions, such as ITS and 18S (Verweij and Stensvold, 2014; Hii et al., 2018), but increased availability of whole-genome sequence data is facilitating the discovery of more sensitive and species-specific genomic targets (Pilotte et al., 2016a; Papaiakovou et al., 2017).

Repetitive elements are essential parts of eukaryotic genomes that have structural and regulatory functions (Shapiro and Sternberg, 2005; López-Flores and Garrido-Ramos, 2012), and different types of repetitive DNA elements have been studied and classified (Charlesworth et al., 1994; Plohl et al., 2008; López-Flores and Garrido-Ramos, 2012; Biscotti et al., 2015). Ribosomal DNA is found in repeat arrays (Lafontaine and Tollervey, 2001; Pruesse et al., 2007). These have traditionally been used for primer design and can give sensitive results depending on the size of the array (Verweij and Stensvold, 2014). However, the repeat is oftentimes conserved between species and even genera, and rDNA-based assays are often less specific than those designed from other repeat types (Pilotte et al., 2016; O’Connell et al., 2018).

Tandemly repeated DNA is classified by the size of the repeated monomer, resulting in microsatellites (< 9 bp), minisatellites (< 15 bp in arrays of 0.5–30 kb), and satellites (satDNA, up to ∼200 bp per monomer, in megabase-sized arrays) that are generally enriched within the centromeric, pericentromeric, and subtelomeric regions of the chromosome (López-Flores and Garrido-Ramos, 2012). Copy number can be quite variable in mini- and micro-satellites but larger satDNA monomers are more consistent within species (López-Flores and Garrido-Ramos, 2012). Microsatellites and minisatellites are not useful for assay design, as the repeats tend to be too short to allow for primer/probe design. Larger satDNA monomers, on the other hand, offer the best options for assay design, in that the repeat monomers are an optimal size for qPCR, they are extremely abundant, and the copy number is relatively stable within species (López-Flores and Garrido-Ramos, 2012).

Other types of repetitive DNA include transposons and retrotransposons, which are dispersed throughout the genome. These include short interspersed nuclear elements (SINEs) which are 100 to 500 bases long, and long interspersed nuclear elements (LINEs) which are larger—6,000 or 7,000 bases long. These may also be useful for repeat-based assay design (Funakoshi et al., 2017).

The amount of repetitive DNA in any eukaryotic species is variable and can make up quite a large percentage of the genome. A recent study of parasitic worms revealed that both genome size and repeat content of the genomes range widely, with repeat elements forming up to 37% of the genomes in STH of interest (**Table 1**). Repetitive elements make up even greater percentages in other eukaryotes (Brindley et al., 2003; Wickstead et al., 2003; Shapiro and Sternberg, 2005; Coghlan et al., 2019), up to an astonishing 97% in some plants (Flavell et al., 1974; Piednoël et al., 2012). These repetitive elements, because of their abundance in the genome, provide targets for molecular assays of exquisite sensitivity. In addition, since many appear unbound by selective pressure, they can be highly species-specific. This combination of improved sensitivity and species-specificity makes repetitive elements a prime target for molecular diagnostics. However, as mentioned above, some forms of repetitive DNA, such as simple short nucleotide repeats, will be unsuitable for assay design. Another potential stumbling block is sequence variation in the repeat itself as polymorphism in the primer and probe sites will decrease the sensitivity of the assay. However, although the repeats we have targeted have not been specifically investigated, maintenance of homogeneity in repetitive elements by concerted evolution has been discussed in relation to other repeats in other species (Ganley and Kobayashi, 2007; Teruel et al., 2014). Concerted evolution of repeats conserves the sequence within a species while allowing significant heterogeneity between species. There can, of course, be some variation within repeats (Onyabe and Conn, 1999; Lindner and Banik, 2011) which could lessen the sensitivity of repeat-based molecular assays. Copy number variation of repeats between individuals may also impact the sensitivity of these assays in some populations. Studies have found population-level copy number variation in ribosomal repeats of different species (Bik et al., 2013; Schaap et al., 2013; Mascagni et al., 2018; Zhao and Gibbons, 2018), but there is a suggestion that there are both copy number variable-type repeats and constant-type repeats, whose copy number is consistent within species (Umemori et al., 2013). An understanding of copy number variation and its impact on assay sensitivity will likely need to be studied on an individual species-by-species basis.

**Table 1:** Genome size of representative helminth species and the amount/percent of genome masked as repetitive. Low complexity repeats and simple (for example di- or tri-nucleotide repeats) are not targets for molecular assays.

Species	Assembly size (Mb)	Repeat-masked (Mb)	Repeat %	% of assembly that is low complexity/simple repeats
*Ancylostoma ceylanicum* (Hii et al., 2018)	349	128.6	36.8	0.5
*Ancylostoma duodenale* (Hii et al., 2018)	332.9	116.1	34.9	0.7
*Ascaris lumbricoides* germline (Okorie and de Souza, 2016)	334	n/a	16.8	n/a
*Ascaris lumbricoides* soma (Okorie and de Souza, 2016)	291	n/a	7	1.5
*Necator americanus* (Masny et al., 2016; Hii et al., 2018)	244.1	67.1	27.5	1.2
*Strongyloides stercoralis* (Pilotte et al., 2017; Hii et al., 2018)	42.7	4.4	10.3	4.2
*Trichuris trichiura* (Hii et al., 2018)	75.5	18.4	24.4	0.4

The value of repeats as diagnostic tools has been understood for some time but these sequences were more difficult to find in the pre-genomics era (McReynolds et al., 1986; Meredith et al., 1989; Chanteau et al., 1994; Nekrutenko et al., 2000; Hamburger et al., 2001; Rao et al., 2002; Demas et al., 2011; Lodh et al., 2016). Now, abundant genomic data and robust bioinformatics tools are available to make these targets easier to identify and use in developing PCR-based assays. The pipeline leading from low coverage NGS data to hyper-sensitive and specific qPCR assay is not overly complicated or time-consuming, and the ability to repurpose low coverage NGS data from other studies makes this an attractive option for diagnostic development for helminthology and many other fields.

## Findings

Diagnostics give an estimate of the true prevalence of a disease, with the probability of correctly estimating the truth given by the sensitivity and species-specificity of the diagnostic. WHO guidelines for when to treat a community are informed by the prevalence of disease in that community, and are thus influenced by the sensitivity of the diagnostic used. A study modeling the probability of making the correct treatment decisions given WHO guidelines for treatment and varying the true prevalence and diagnostic sensitivity shows that there is a significant difference in outcome when more sensitive diagnostics are used (Medley et al., 2016). Medley et al. (Medley et al., 2016) found it especially true in areas of intermediate true prevalence (between 30% and 50%). More sensitive tests allowed the correct treatment decision to be made more often in intermediate cases.

The aforementioned study measured outcome by looking at DALYs and found that these were not as influenced by diagnostic sensitivity in low or high prevalence areas. However, there are other reasons to prefer more sensitive tests in low prevalence areas. As the goals of the WHO elimination programs are reached, there will be pressure to reallocate the funds spent on MDA to other programs. A well-defined threshold under which recrudescence will not occur is critical to preventing the reoccurrence of disease after the completion of MDA. Restarting such programs would be extremely difficult and expensive once they have ended. Modeling has shown that the threshold must be based on true prevalence (Truscott et al., 2017; Ásbjörnsdóttir et al., 2017), which can only be accurately estimated with highly sensitive and species-specific diagnostics. Improved diagnostics are crucial to meet this need (Kongs et al., 2001; Andersen et al., 2013; Clarke et al., 2018; Hidalgo et al., 2018). Post-MDA surveillance is also necessary. With highly sensitive diagnostics, the reappearance of disease can be recognized and addressed before infection levels rise, increasing the probability of controlling the recrudescence (Farrell and Anderson, 2018). In addition to testing human populations for infection, vectors that transmit helminths (or other parasites) or intermediate hosts can be screened to track disease prevalence in the community without the need for taking human samples (Sanpool et al., 2012; Pilotte et al., 2016b; Ramírez et al., 2018; Zaky et al., 2018). Pooling of samples is a common way to decrease cost but diagnostic tests used for the screening of pooled samples need to be highly sensitive (Masny et al., 2016; Okorie and de Souza, 2016; Pilotte et al., 2017), especially when the infection level is low, so as not to miss positive results in dilute samples.

**Figure 1:**
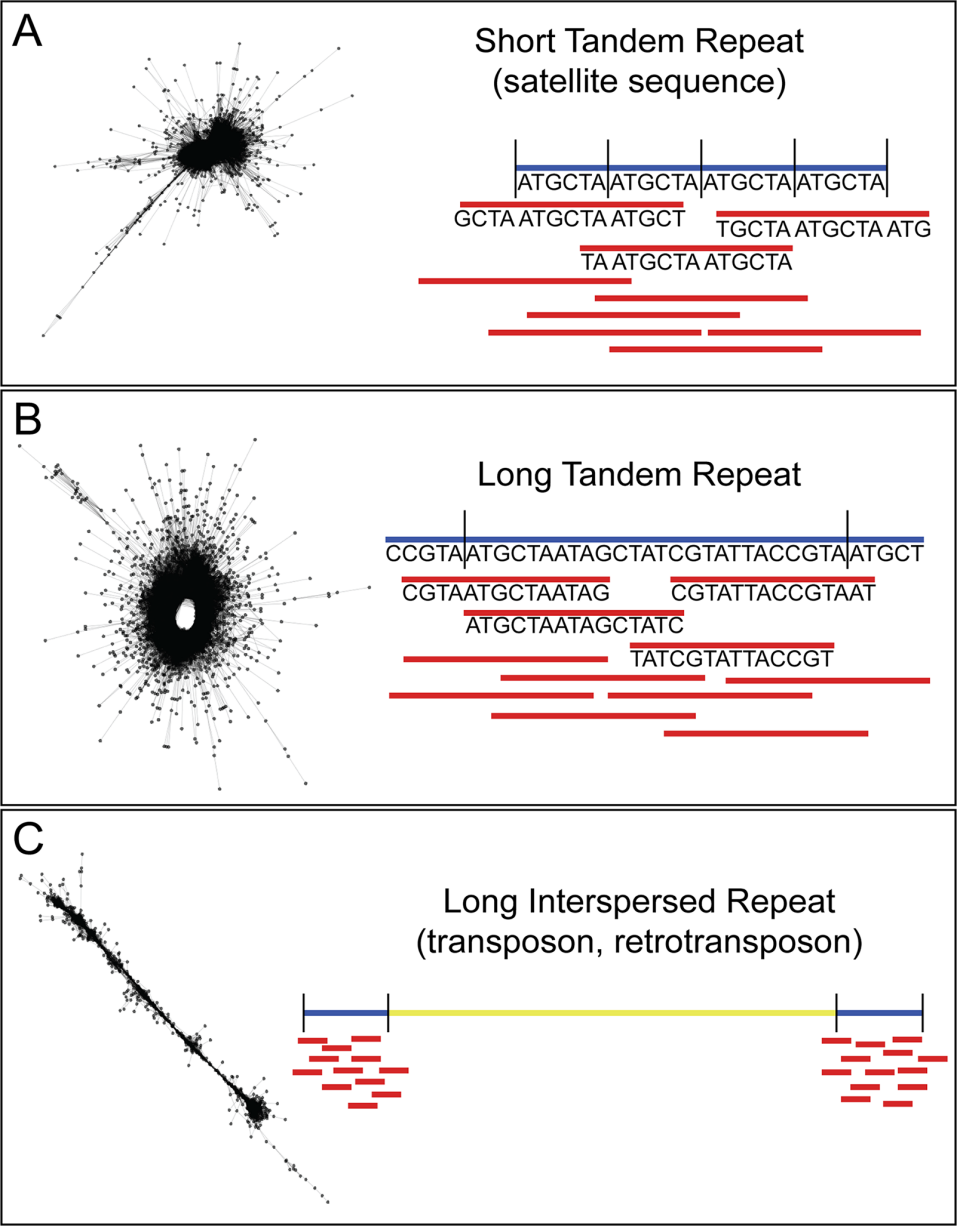
Description of RepeatExplorer cluster output. **(A)** Short tandem repeats, including many satellite sequences, form characteristic “star burst”-type clusters. Because they are tandem, and such repeats are of similar size or shorter than the length of an individual sequence read, a very high percentage of reads within the cluster meet the RepeatExplorer-defined criteria for pair formation. This results in each read successfully pairing with a very high percentage of the other reads assigned to the cluster. Because nearly all of the reads within the cluster are paired with nearly all of the other reads within this same cluster, a compact network of very short edges forms between reads. This in turn generates a very dense cluster with a core of paired reads possessing nearly identical sequences. If of sufficient length for assay design, the consensus sequences for these clusters make ideal diagnostic targets, as they contain the greatest number of repeats per read. **(B)** Long tandem repeats characteristically result in “doughnut”-like clusters. In such clusters, neighboring reads within the underlying scaffold meet the criteria for pair formation. However, because the length of the repeat monomers generating these clusters is longer, reads may be significantly shorter than the monomer itself. This results in many reads within the cluster that do not meet the criteria for pair formation as they map to different regions of the same monomer unit. Yet because they are tandemly arranged, reads spanning a repeat-repeat junction will meet the pair formation criteria, closing the sequence “loop” and resulting in a “doughnut”-shaped cluster. **(C)** Long interspersed repeats, such as transposable elements form characteristic “line”-type clusters. While reads neighboring each other in the underlying scaffold meet the criteria for pair formation, the extended length of a repeat monomer means that distant reads within a single monomer will not meet this threshold. This results in similar pairings to those seen in clusters generated from long tandem repeats. However, because these elements are interspersed, reads do not span repeat-repeat junctions, so a “loop” is not formed, and clusters attain a linear appearance.

In recent years, efforts to sequence nematode genomes by groups, such as the International Helminth Genomes Consortium, have made great strides in increasing the availability of helminth genomic sequence data (Howe et al., 2016). Although many of the genomes are in draft form, this is sufficient for probing the genome for species-specific repeats. Our method for recovering highly repetitive sequence from low depth, raw, short-read genome sequence data uses the Galaxy-based tool RepeatExplorer (Novák et al., 2010; Novák et al., 2013; Pilotte et al., 2016a). Originally used to investigate repeat sequences in plant genomes, RepeatExplorer takes as input short-read next-generation sequence data and creates graph-based clusters based on the similarity of the sequences. In these graphs (see **Figure 1**), each read is represented by a node, and each sequence pair (by default defined as ≥90% identity over at least 55% of the read lengths) is represented by an edge. The density of the graph represents the number and similarity of reads in the cluster. In low-depth sequence data sets, low copy number sequences will not be well represented and will, therefore, graph as individual nodes or small clusters, whereas high copy number repetitive sequences will be found in dense clusters. The number of reads in a given cluster, combined with the structure and density of that cluster, can be used as a proxy for the representation of the number of repeats of that sequence in the genome. The copy number of the repeat will impact the sensitivity of the assay, since each copy in the genome will be an additional target for the assay. In addition, RepeatExplorer and its sister software TAREAN (Novák et al., 2017) provide information on the count of individual nucleotides in the repeat contigs. These counts can be used to design assays to the most conserved regions, limiting the problems associated with intragenomic variation. We have developed, and made available here, custom Python scripts to parse the output from RepeatExplorer and return highly repetitive sequences (https://github.com/JessicaGrant/RepeatTargetScripts). Primer/probe qPCR assays targeting sequences discovered using this technique have been shown to amplify as little as 20 ag of genomic DNA, or less than the amount of DNA found in a single egg (Pilotte et al., 2016a). Care is needed in choosing which repeat to select for use as a diagnostic target, as some may be found in closely related species (Williams et al., 2000; Rao et al., 2006); however, similar to what has been reported in the literature (Subirana and Messeguer, 2013; Subirana et al., 2015), we have found that many repetitive sequences are species-specific. There may be times when a more general assay—one that will amplify several species of the same genus, for example—may be desired (Rao et al., 2006). A careful search of RepeatExplorer output can often reveal both species- and genera-specific targets.

Diagnostics based on targets discovered using this technique have proven useful in both past and ongoing field tests in Kenya (Pickering et al., 2019), Bangladesh (Benjamin-Chung et al., 2019), Ethiopia, Uganda, Timor Leste (Papaiakovou et al., 2017), Thailand (O’Connell et al., 2018), Liberia (Fischer et al., 2018), Japan, Benin, Malawi, India, and the Southern US, and have been adopted for use by large operational research efforts, such as the DeWorm3 cluster randomized trials (Ásbjörnsdóttir et al., 2018). However, testing biological samples, whether for diagnosing individuals or getting an overview of the epidemiological environment of a region, involves a myriad of factors, such as unexpected or emerging parasites, zoonotic infections, and misleading material in the samples. Although most of the criticism of the KK technique has been on the lack of sensitivity and potential for missed infection, there is also the risk of false positives, for example, mistaking other material in stool as eggs (Speich et al., 2015). Some fecal elements may resemble parasite ova, depending on environmental or dietary factors. Confounding elements may include pollen grains, fungal spores, diatoms, or any number of items. An entire chapter in Ash and Orihel’s “Atlas of Human Parasitology” is dedicated to artifacts in fecal samples that can mislead copromicroscopic diagnostics (Cushion et al., 1990). Thus, this problem is more frequent than many researchers realize or acknowledge; what follows are some examples demonstrating the importance of this issue.

A field study comparing KK with repeat-based qPCR in Bangladesh (Benjamin-Chung et al., 2019) found that hookworm species and *Trichuris trichiura* prevalence, as measured by qPCR, was significantly higher than was measured by KK. This was expected, given the greater sensitivity of the qPCR assays and the results of many previous studies comparing KK and PCR (Pontes et al., 2003; Stensvold et al., 2006; Knopp et al., 2014; Pilotte et al., 2016a; Easton et al., 2017; Ng-Nguyen et al., 2017). For *Ascaris*, however, prevalence as measured by KK was significantly higher than by qPCR. This surprising result was investigated further, both by qPCR targeting a different part of the *Ascaris* genome, and also by amplicon sequencing that targeted the 18S gene of all eukaryotes in several of the KK positive/qPCR negative samples. All of the samples that were positive by KK but negative by the initial qPCR assay were also negative using the second qPCR target. Additionally, the 18S amplicon sequencing revealed no *Ascaris* in these samples, but did find it in the control samples that were positive by both KK and qPCR. Not one organism was found in the amplicon sequencing that could explain all of the false-positive results. What material in the samples had confounded the microscopists is still unknown, but there was no evidence by any of the molecular assays that *Ascaris* was present in the samples. Had the study relied on copromicroscopic results alone, the conclusion would have been that MDA or WASH interventions were less effective as an *Ascaris* intervention than they likely were, since the true prevalence of the parasite was in fact much lower than was measured by KK.

In a similar case, higher than expected rates of hookworm were noted in a survey of children in rural Rwanda. Further investigation suggested these results may have been confounded by *Caenorhabditis elegans* eggs (Irisarri-Gutiérrez et al., 2016). Additional examples of misidentification of hookworm ova as other eggs (Ralph et al., 2006; Werneck et al., 2007; Yong et al., 2007) show that such confusion may be a more common problem than previously thought. Thus, relying solely on microscopy may be misleading in some instances.

Discrepancies can occur between molecular assays as well, since some PCR targets are less species-specific than others. In developing our pipeline for repeat-based primer discovery, a previously published qPCR assay targeting the internal transcribed spacer region was compared against our newly developed assay targeting an *Ancylostoma duodenale* species-specific repeat (Llewellyn et al., 2016; Pilotte et al., 2016a). Surprisingly, the repeat-based assay failed to detect any of the samples that were determined to be positive for *A. duodenale* by the ITS-based assay. A previously published semi-nested PCR assay (George et al., 2015; Chidambaram et al., 2017) and Sanger sequencing later determined that the discordant results were due to all of the infections being the zoonotic species *Ancylostoma ceylanicum*. The ITS of these two species is highly conserved in the region targeted by the original qPCR assay and so the ITS-based assay did not distinguish between these closely related species. Our repeat-based *A. duodenale* assay, on the other hand, only detects *A. duodenale*, and so all of the *A. ceylanicum*-containing samples were negative. We have since used our pipeline to develop a species-specific qPCR assay for *A. ceylanicum* (Papaiakovou et al., 2017), which is more sensitive and specific than the ITS-based assay and easier to use than semi-nested PCR.

In a similar case, the same ITS-based primer set (Llewellyn et al., 2016) was used to investigate *Ancylostoma duodenale* in a field study of a refugee population in Thailand (O’Connell et al., 2018). Since the most common human *Ancylostoma* parasite is *A. duodenale*, the results were initially believed to indicate *A. duodenale* infection. Again, however, a corroboratory qPCR targeting the highly specific *A. duodenale* repeat failed to detect any *A. duodenale*. Use of the *A. ceylanicum*-specific assay (Papaiakovou et al., 2017), as well as confirmation with semi-nested PCR and Sanger sequencing, revealed that all of these infections were, in fact, caused by *A. ceylanicum* and not *A. duodenale*. In this case, although the more general ITS-based assay misdiagnosed the species causing the infection, the more specific assay for the expected parasite (*A. duodenale*) would have missed the infection. This highlights a risk of using extremely specific qPCR assays in the field if the precise parasite community is unknown. It also highlights that *A. ceylanicum* may be a much more common human pathogen than previously supposed. Here, the repeat-based, species-specific assays can be used to identify the true prevalence of various related parasite species.

The specificity of *Trichuris trichiura* (whipworm) detection by microscopy is assumed to be high, given the relatively distinct morphology of *Trichuris* eggs. However, there are several species of *Trichuris*, including some that infect companion or farm animals. Distinction between species of *Trichuris* relies on size differentiation, but there are overlaps in some species making misdiagnosis by microscopy possible. The most common species in humans is *T. trichiura*, but *Trichuris suis*, which commonly infects pigs, and *Trichuris vulpis*, usually found in dogs, have also been found infecting humans (Areekul et al., 2010; Mohd-Shaharuddin et al., 2019). Microscopy and genera-specific qPCR assays may be confounded by these zoonotic species. Discordance between the ITS-based *Trichuris* qPCR assay and the repeat-based qPCR assay has been noted in one study where all but one of the discordant results were later shown to be *Trichuris ovis*, a species found in sheep and goats that has not been known to infect humans (Pilotte et al., 2016a). Whether this is evidence of human infection or merely false positives due to close contact with infected animals or ingesting food contaminated with animal feces is an open question. In these cases, qPCR targeting highly species-specific repetitive targets alone could easily miss the prevalence of zoonotic infection, leading to a misunderstanding of the health of the population. On the other hand, the use of the repeat-based, highly specific assays gives a true picture of the prevalence of various species.

Despite the distinct morphology of *Trichuris*, the potential of misidentifying unexpected infection with zoonotic species is not the only risk when relying solely on microscopy for investigating whipworm. A study of STH in two regions in Liberia that used both microscopy and qPCR found discordance between the tests for *T. trichiura* in one of the regions (Fischer et al., 2018), with 25 of 27 putatively positive samples for *T. trichiura*, as determined by KK, being negative by qPCR. In the second region, the agreement between the two tests was high. In this case, the discrepancies were investigated further, and the eggs were determined to be a species of *Capillaria*, a human parasite that is associated with eating raw fish and was not expected to be found in this region. Reinvestigation by microscopy in this case elucidated subtle differences in the eggs found in the KK-positive, qPCR-negative samples. A microscopist, highly trained and expecting to have to differentiate between two extremely similar egg morphologies, could have noticed the difference and provided a correct result. However, since *Capillaria* had not previously been reported in Liberia, the eggs that looked like *Trichuris* were reported as such. Without the complementary qPCR, this mistake would not have been discovered. This study also found discrepancies between the microscopy and qPCR measuring the prevalence of *Ascaris lumbricoides* in the same region where *Capillaria* was discovered. Surprisingly, these samples were also determined to be confounded by the presence of *Capillaria*, which can appear rounder and have more subtle polar plugs, leading to its misidentification as the eggs of *A. lumbricoides*. These examples highlight the tendency for microscopists to sometimes see what they are looking for. In this case, the unexpected presence of *Capillaria* eggs misled the microscopists and would have resulted in significant misinterpretation of the distribution of STH species in the study region.

## Conclusion

The elimination of STH is a worthy goal, given the distress and disability they cause to a large portion of the global population. The goal is attainable, but will not be easily reached. Monitoring and evaluation of progress is critical and depends on highly accurate reporting. Species-specific repeat-based target discovery and qPCR assays deliver this accuracy. Microscopy and genera-specific molecular assays have a place in this effort, especially in surveys where full mapping of parasite diversity has not occurred. These tools, used in conjunction with highly sensitive and specific molecular assays targeting repetitive elements, can give a clear and accurate assessment where one tool alone could yield misleading results.

New target development is fairly easy, since the web-based bioinformatics tool RepeatExplorer provides output in a manner that makes finding repeat-based qPCR targets straightforward. Only a skim of the genome is necessary, so the data needed to develop new assays is already available for many species and is fairly inexpensive to produce for a species whose genome has not yet been sequenced. We have used this technique to explore new diagnostics for other nematode and protist parasites, and we think that the pipeline for assay development has potential for improving diagnostic sensitivity for many other classes of infectious agents.

Correct identification of species is of interest if one wants to understand evolution, biogeography, and emerging disease. The treatment for infection with one species of helminth is often the same as for another; however, we would argue that lumping all species of a genus together is careless. It is not clear that different species respond the same way under the same drug treatment, and misidentification of species in the past might be confounding the results of studies of resistance or drug response based on microscopy alone. Different species within a genus may also vary in their capacity for animal infection, resulting in some parasites having animal reservoirs while others remain obligate human pathogens. A more detailed understanding of the underlying community structure will offer crucial insight into subjects, such as antihelminthic resistance, emerging or zoonotic diseases, and optimal threshold levels for elimination of disease.

## Bioinformatic Resources

RepeatExplorer (Novák et al., 2010; Novák et al., 2013) is made freely available here:

http://repeatexplorer.org/

We make our scripts available for others to adapt and use here:

https://github.com/JessicaGrant/RepeatTargetScripts

## Author Contributions

Conceptualization: JG, NP, and SW. Funding acquisition: SW. Writing—original draft: JG. Writing—review and editing: JG, NP, SW.

## Funding

This work received financial support from the Coalition for Operational Research on Neglected Tropical Diseases (CORNTD), which is funded at The Task Force for Global Health primarily by the Bill & Melinda Gates Foundation, by the UK aid from the British government, and by the United States Agency for International Development through its Neglected Tropical Diseases Program. In addition, this work was partially funded through a grant to the DeWorm3 Project, which is funded by a grant to the Natural History Museum from the Bill and Melinda Gates Foundation.

## Conflict of Interest Statement

The authors declare that the research was conducted in the absence of any commercial or financial relationships that could be construed as a potential conflict of interest.
